# Clinical practice guideline monitoring children and young people with, or at risk of developing autosomal dominant polycystic kidney disease (ADPKD)

**DOI:** 10.1186/s12882-019-1285-2

**Published:** 2019-04-30

**Authors:** Jan Dudley, Paul Winyard, Matko Marlais, Oliver Cuthell, Tess Harris, Jiehan Chong, John Sayer, Daniel P. Gale, Lucy Moore, Kay Turner, Sarah Burrows, Richard Sandford

**Affiliations:** 1 University Hospitals Bristol NHS Foundation Trust, Bristol, UK; 2 University College London Medical School, London, UK; 3 Plymouth Hospitals NHS Trust, Plymouth, UK; 4 Polycystic Kidney Disease Charity, London, UK; 5 University of Sheffield, Sheffield, UK; 6 Newcastle University, Newcastle, UK; 7 Queen Elizabeth Hospital Birmingham, Birmingham, UK; 8 Addenbrooke’s Hospital, Cambridge, UK; 9 Patient Representative, c/o The Renal Association, Bristol, UK

## Abstract

Autosomal Dominant Polycystic Kidney Disease (ADPKD) is thought to affect about 1 in 1000 people in the UK. ADPKD causes a progressive decline in kidney function, with kidney failure tending to occur in middle age. Children and young people with ADPKD may not have any symptoms. However they may have high blood pressure, which may accelerate progression to later stages of chronic kidney disease.

There is uncertainty and variation in how health professionals manage children and young people with confirmed or a family history of ADPKD, because of a lack of evidence. For example, health professionals may be unsure about when to test children’s blood pressure and how often to monitor it in the hospital clinic or at the GP. They may have different approaches in recommending scanning or genetic testing for ADPKD in childhood, with some recommending waiting until the young person is mature enough to make this decision his or herself.

This guideline is intended to help families affected by ADPKD by making sure that:

- health professionals with specialist knowledge in ADPKD offer you information on inheritance and potential benefits and harms of testing for ADPKD.
- the decision to test and the method of testing for ADPKD in children and young people is shared between you or your family and the health professionals
- blood pressure assessment is undertaken regularly in children and young people at risk of developing ADPKD

## Method used to arrive at a recommendation

Evidence reviews were undertaken that focused on literature in relation to children and young people (CYP) with, or are at risk of developing Autosomal Dominant Polycystic Kidney Disease (ADPKD). Medline (1980 – December 2017), EMBASE (1980 – December 2017) and PsychInfo databases were searched as well as websites of national associations in this field. A search strategy was developed by the guideline committee to ensure that all papers addressing the questions were identified using search terms based on PICO methodology (Table 1). The clinical leads also hand searched reference lists of reviews and included papers.

**Table 1:** PICO characteristics

Population	Intervention	Comparison	Outcome	Study design
Children (< 18) with a confirmed diagnosis of ADPKD or at risk of ADPKD due to their family history	Ultrasonography Cranial imaging Blood Pressure monitoring Monitoring for albuminuria Therapeutics Genetic counselling	Any intervention compared with any other or no intervention	Mortality Hospitalisations Chronic Kidney Disease Cardiovascular disease Hypertension	Randomised controlled trials (RCT) Non-randomised studies if adjusted for key confounders (age, health at baseline, co-morbidities).

Abstracts were screened for relevance by a clinical lead and 1 other member of the guideline committee according to pre-defined inclusion and exclusion criteria as detailed in the scope. Abstracts identified for review by the two reviewers were compared and any disputed abstracts were resolved by the guideline committee. The full papers were then reassessed by the clinical lead to further exclude any study that does not meet the following predefined criteria: Randomised controlled trials (RCT), non-randomised studies if adjusted for key confounders (age, health at baseline, co-morbidities).

Clinicians on the guideline committee critically appraised any eligible papers using critical appraisal skills programme tools [1]. Where evidence was lacking, formal Delphi consensus methodology was employed. A Delphi panel was constituted, comprising representation from each specialist area covered by the guideline: Nephrology services (3 adult and 3 paediatric nephrologists), clinical genetics (3 representatives), paediatrics with an interest in nephrology (3 representatives), lay members (3) and general practitioners (3 invited, 2 responded). A Likert scale was used for panellists to provide their responses to statements. Consensus agreement and disagreement was defined as 80% of panellists selecting ‘agree’ or ‘disagree’ respectively. Individual responses were anonymised to panellists and the working group, with the exception of the chair. No literature was sent to participants to avoid risk of bias. The process was iterative (participants able to change their views in subsequent rounds). Three rounds were undertaken.

## Background

This guideline makes recommendations for monitoring children and young people (CYP) up to 18 years of age with, or at risk of developing Autosomal Dominant Polycystic Kidney Disease (ADPKD).

ADPKD is the commonest inherited renal disease with an incidence of around 1 in 1000 and accounts for 5–7% of adults commencing renal replacement therapy [2, 3]. Whilst ADPKD has traditionally been thought of as an adult disease, with established renal failure tending to occur in or after the 6th decade, there is clear evidence of earlier manifestation in children and young people (CYP), in whom hypertension and proteinuria may accelerate progression to later stages of chronic kidney disease. There is wide variation in clinical practice facing CYP with confirmed or a family history of ADPKD, with regard to a) assessment of blood pressure and urine testing for the presence of proteinuria b) ultrasound testing to evaluate presence of cysts and c) genetic counselling and testing. In order to improve quality of care and reduce variation in practice, the British Association for Paediatric Nephrology (BAPN) and the UK Renal Association (RA) in collaboration with key partners, has undertaken this work to develop best practice guidance in the area.

## Summary of clinical practice guidelines

### Guideline 1

We recommend that parents or carers of children at risk of developing ADPKD should be offered information on ADPKD inheritance and potential benefits and harms of testing for ADPKD, by health professionals with specialist knowledge in this area. (1D).

### Guideline 2

We recommend that children and young people aged 5 years and above with, or at risk of developing ADPKD, should have an assessment of blood pressure (BP) at least once every 2 years. (1B).

### Guideline 3

We recommend that the decision to test for ADPKD in asymptomatic children and young people (CYP) at risk of developing ADPKD, should be undertaken jointly between health professionals and parents or carers and, wherever possible, the young person. (1D).

### Guideline 4

If testing is decided on, we suggest that either kidney ultrasound or genetic testing may be offered to asymptomatic children and young people at risk of ADPKD, where testing has been agreed by parents or carers (and, wherever possible, the young person) and health professionals (2D).

### Guideline 5

We suggest that, if asymptomatic children at risk of ADPKD do not have cysts on ultrasound, further ultrasound testing should be deferred until adolescence (15–18 years), or later if preferred by the young person (2D).

### Guideline 6

We recommend that if genetic testing is planned in children and young people at risk of ADPKD, identification of the mutation in the affected adult family member (if not already known) should be undertaken prior to testing the child or young person. (1D).

## Summary of audit measures

- **Audit Measure 1:** Proportion of parents or carers of children at risk of developing ADPKD offered information on ADPKD inheritance and potential benefits and harms of testing for ADPKD
- **Audit Measure 2:** Proportion of children and young people aged 5 years and above with, or at risk of developing ADPKD, having an assessment of blood pressure (BP) at least once every 2 years
- **Audit Measure 3:** Proportion of asymptomatic children and young people at risk of developing ADPKD offered testing for ADPKD
- **Audit Measure 4:**
  - Proportion of asymptomatic children and young people at risk of developing ADPKD offered genetic testing for ADPKD
  - Proportion of asymptomatic children and young people at risk of developing ADPKD offered ultrasound testing for ADPKD
- **Audit Measure 5:** Proportion of asymptomatic children at risk of ADPKD who do not have cysts on ultrasound, having repeated ultrasound testing prior to adolescence (15–18 years)
- **Audit Measure 6:** Proportion of asymptomatic children at risk of ADPKD whose parents have been tested for a genetic mutation prior to the child being tested

## Summary of research recommendations

**Research recommendation 1:** In children and young people with ADPKD, does regular (e.g. yearly or every 2 years) urine albumin: creatinine monitoring and treatment reduce disease progression?

**Research recommendation 2:** In children and young people with ADPKD what is a) the incidence of sub-arachnoid haemorrhage and b) the prevalence of intracranial aneurysm?

**Research recommendation 3:** In adults, children and young people with ADPKD with a family history of intracranial aneurysm or sub-arachnoid haemorrhage does Intracranial Magnetic Resonance (MR) imaging reduce the risk of intracranial events?

## Rationale for clinical practice guidelines

### Guideline 1

We recommend that parents or carers of children and young people at risk of developing ADPKD should be offered information on ADPKD inheritance and potential benefits and harms of testing for ADPKD, by health professionals with specialist knowledge in this area. (1D)

- **Audit Measure 1:** Proportion of parents or carers of children at risk of developing ADPKD offered information on ADPKD inheritance and potential benefits and harms of testing for ADPKD

#### Rationale

No relevant studies were identified for this review question; however, NICE guidance on patient experience in adult NHS services recommends that patients should be provided with information, and the support they need to promote their active participation in care and self-management. This should include information about relevant treatment options and services that they are entitled to, even if these are not provided locally [4]. There was 100% agreement with this recommendation in the Delphi consensus process. Health professionals should be aware of the anxiety suffered by families relating to uncertainty of diagnosis as well as at times of testing for ADPKD.

Health professionals should discuss the limitations of testing modalities for ADPKD. Renal ultrasound cannot effectively exclude ADPKD in children [5]. In a retrospective cohort study of ultrasound assessment in children and young people under the age of 15 at risk of ADPKD, 193/420 CYP were diagnosed with cysts at baseline visit (mean age 8.6 +/− 4.2 years) with 227 having no cysts (8.0 ± 4.1 years). In follow up to age fifteen, 18/77 (23%) of the latter group who underwent repeat ultrasound developed cysts. In other words, 23% of CYP with no cysts visible on initial renal ultrasound who received a further ultrasound, were diagnosed with ADPKD [6]. No standardised criteria for renal ultrasound diagnosis of ADPKD exist for children under the age of 15 years, and even below the age of 30, there is a significant false negative rate. This is particularly so in families carrying a *PKD2* mutation, which is typically associated with milder disease. In these families, 16.5% of patients between the ages of 15–29, with no cysts on initial ultrasound scan, go on to a diagnosis of ADPKD later in life [7]. While genetic testing is more definitive, it is most likely to be informative in families of known genotype, as around 10% of families phenotypically affected by ADPKD do not carry a pathogenic mutation of *PKD1* or *PKD2* that is detected by current technologies [8]. Health professionals (generally nephrologists, geneticists or paediatricians with interest in nephrology) providing information on testing should have a good understanding of these issues.

### Guideline 2

We recommend that children and young people aged 5 years and above with, or at risk of developing ADPKD, should have an assessment of blood pressure (BP) at least once every 2 years. (1B).

**Audit Measure 2:** Proportion of children and young people aged 5 years and above with, or at risk of developing ADPKD, having an assessment of blood pressure (BP) at least once every 2 years.

#### Rationale

Low quality evidence identified for this review question was deemed by the committee to be sufficient to make a recommendation without the need for formal consensus. A recent systematic review of 928 children with ADPKD across 14 studies estimated the prevalence of hypertension to be 20% (95% CI 15–27%) [9]. There is also evidence to suggest that children with ADPKD whose BP is high or borderline high have an increased left ventricular mass index on echocardiography, a marker of cardiac target organ damage [10]. Studies have also shown that children with ADPKD who are hypertensive show an increased total kidney volume, compared to those that are normotensive [11–13]. There are, as yet, no studies assessing long term outcomes for the treatment of hypertension in children with ADPKD. However, there is clear evidence of benefit of treating hypertension in children generally and especially in those with chronic kidney disease [14].

We recommend that both children with a confirmed diagnosis of ADPKD and those at risk of ADPKD through family history should have BP monitored, since some families may choose not to undertake testing for ADPKD in childhood. The risk of developing hypertension in children with ADPKD rises with age [9]. Hypertension under the age of 5 years is uncommon in ADPKD [15] and should prompt a search for an alternative diagnosis. In the absence of any other disease process BP rises slowly in children with ADPKD, therefore monitoring BP every 2 years should be sufficient to detect a rise in BP requiring treatment during childhood.

### Guideline 3

We recommend that the decision to test for ADPKD in asymptomatic children and young people (CYP) at risk of developing ADPKD, should be undertaken jointly between health professionals and parents or carers and, wherever possible, the young person. (1D).

**Audit Measure 3:** Proportion of asymptomatic children and young people at risk of developing ADPKD offered testing for ADPKD.

#### Rationale

No relevant studies were identified for this review question. There was 88% agreement with this recommendation in the Delphi consensus process.

Inheriting and passing on a genetic disease may have significant psychological impact, ranging from frank depression through anxiety, guilt, anger, uncertainty and sadness [16]. This genetic anxiety or ‘guilt’ may increase with uncertainty over disease variability and where there is a perceived lack of diagnosis or effective therapy. These are common issues in CYP with ADPKD, which may be further compounded by mixed messages from the medical community, with conflicting opinions as to the clinical significance of ADPKD in childhood. Genetic Counselling and testing for a condition in at risk individuals has been reported to ameliorate the psychological burden for some families and children [17–19], however, wherever possible, the child or young person should be involved in this decision. Article 12 of the United Nations Convention on the Rights of the Child (1989) states that we must assure ‘the child who is capable of forming his or her own views has the right to express those views freely’ and that ‘the views are given due weight in accordance with the age and maturity of the child’ [20].

Important issues to be discussed in counselling before deciding to test for ADPKD include:

- Age and ‘Gillick’ competence of the CYP and consideration of whether to wait until they can make a fully-informed decision.
- Risk of false negative results for ultrasound and genetic tests as described on page 9
- Implications for ongoing management. Blood pressure monitoring is likely to be as important as making the diagnosis in preventing long term complications, but this should be balanced against the psychological benefits of confirming / refuting (noting risk of false negative results) ADPKD. The discussion is likely to change when new treatments become available for children and young people with ADPKD.

A recent European ADPKD Forum multidisciplinary position statement states that ‘Individuals with ADPKD should have access to lifelong, multidisciplinary, specialist and patient-centred care, with information and support to help patients and their families act as fully informed and active partners in care [21].

### Guideline 4

If testing is decided on, we suggest that either kidney ultrasound or genetic testing may be offered to asymptomatic children and young people at risk of ADPKD, where testing has been agreed by parents or carers (and, wherever possible, the young person) and health professionals (2D).

**Audit Measure 4:**

- Proportion of asymptomatic children and young people at risk of developing ADPKD offered genetic testing for ADPKD
- Proportion of asymptomatic children and young people at risk of developing ADPKD offered ultrasound testing for ADPKD

#### Rationale

No relevant studies comparing outcomes in CYP at risk of ADPKD undergoing genetic versus ultrasound testing for ADPKD were identified for this review question. There was 70% agreement with this recommendation (statement) by the Delphi panellists, i.e. consensus not reached. Areas for disagreement largely related to concerns about the utility of ultrasound, particularly in younger children with no standardised criteria for renal ultrasound diagnosis of ADPKD under the age of 15 years and significant false negative rates [22]. The importance of involving CYP in the decision making process was also noted in the Delphi responses. The committee believed that to offer genetic but not ultrasound testing to asymptomatic children and young people at risk of ADPKD could result in a significant change in practice in the absence of evidence and that the importance of individual choice between the two testing modalities should be emphasised given the lack of clear superiority of one over the other. The committee also noted that, although not reaching consensus, the majority (70%) of panellists supported offering a choice of kidney ultrasound or genetic testing to asymptomatic CYP at risk of ADPKD, where testing has been agreed by parents or carers and health professionals (and, wherever possible, the young person) and agreed to make a weaker ‘we suggest’ recommendation.

### Guideline 5

We suggest that, if asymptomatic children at risk of ADPKD do not have cysts on ultrasound, further ultrasound testing should be deferred until adolescence (15–18 years), or later if preferred by the young person (2D).

**Audit Measure 5:** Proportion of asymptomatic children at risk of ADPKD who do not have cysts on ultrasound, having repeated ultrasound testing prior to adolescence (15–18 years).

#### Rationale

No relevant studies assessing outcomes in asymptomatic children at risk of ADPKD undergoing repeated ultrasound testing for ADPKD, compared with those not undergoing repeated ultrasound testing, were identified for this review question. There was 70% agreement with this recommendation (statement) by the Delphi panellists, i.e. consensus not reached. Areas for disagreement largely related to the age of the child at the time of the first negative ultrasound scan and a preference to perform more regular ultrasound scans if requested by parents. There may also be concern by health professionals about the risk of missing very early onset ADPKD, however, these children comprise less than 1% of cases and are likely to be genetically distinct from typical ADPKD, perhaps with multiple compound mutations combining to generate such an aggressive phenotype [23, 24]. This recommendation does not apply to these children, who undergo diagnostic testing for symptomatic disease (e.g. severe renal enlargement, hypertension) and are found to have radiological abnormalities at presentation.

Repeated ultrasound testing is not routinely undertaken in adults with ADPKD where significant progression usually occurs over decades. In the absence of evidence of benefit of repeated ultrasound testing in asymptomatic CYP, together with its resource implications, and the importance of considering the wishes of the young person in the decision making process where possible, the committee was of the view that a recommendation for repeated ultrasound testing prior to adolescence could not be made. The committee considered whether repeated ultrasound testing might help to alleviate parental anxiety, but noted in their experience that repeated ultrasound testing may also add to the psychological burden for families.

The committee also noted that, although not reaching consensus, the majority (70%) of panellists supported deferring further ultrasound testing until adolescence (15–18 years, or later if preferred by the young person) in asymptomatic children at risk of ADPKD who do not have cysts on initial ultrasound. The committee therefore agreed to make a weaker ‘we suggest’ recommendation.

### Guideline 6

We recommend that if genetic testing is planned in children and young people at risk of ADPKD, identification of the mutation in the affected adult family member (if not already known) should be undertaken prior to testing the child or young person. (1D).

**Audit Measure 6:** Proportion of asymptomatic children at risk of ADPKD whose parents have been tested for a genetic mutation prior to the child being tested.

#### Rationale

No relevant studies were identified for this review question. There was 88% agreement with this recommendation in the Delphi consensus process. ADPKD is associated with a wide range of mutations in the genes PKD1 and PKD2, many of which occur only in one family [25]. As a result, genetic testing of PKD1 and PKD2 can find sequence variations that have not been previously described, and it can be difficult to determine the pathogenicity of these changes. First identifying the mutation in the affected adult family member allows for the use of segregation analysis to help assign pathogenicity to any mutations identified in the child or young person. Furthermore, up to 10% of ADPKD is not associated with detectable mutations in PKD1 or PKD2 [8]. This means that failure to identify a PKD1 or PKD2 mutation in a predictive genetic test cannot reliably exclude ADPKD, unless the familial mutation is known be in those genes.

## Rationale for research recommendations

**Research recommendation 1:** In children and young people with ADPKD does regular (e.g. yearly or every 2 years) urine albumin: creatinine monitoring and treatment improve outcome?

### Rationale

The guideline committee was not able to make a recommendation on monitoring of urine albumin: creatinine in CYP with ADPKD or at risk of ADPKD. No relevant studies assessing outcomes in CYP with proteinuria were identified and no consensus was achieved in 2 rounds of the Delphi survey; only 41% of panellists agreed with the statement ‘Urine protein estimation (best assessed at urine albumin:creatinine) should be offered at least every 2 years to children and young people with confirmed ADPKD commencing at 5 years of age’. The prevalence of proteinuria in CYP with ADPKD has been reported to be as high as 20% [9], however, a number of studies have failed to show a relationship between hypertension and proteinuria in children with ADPKD [9, 11, 26]. In adults with ADPKD, established proteinuria and microalbuminuria are reported to be associated with increased mean arterial pressure and more severe renal cystic involvement [27, 28].

**Research recommendation 2:** In children and young people with ADPKD what is a) the incidence of sub-arachnoid haemorrhage and b) the prevalence of intracranial aneurysm?

**Research recommendation 3:** In adults, children and young people with ADPKD with a family history of intracranial aneurysm or sub-arachnoid haemorrhage does Intracranial Magnetic Resonance (MR) imaging reduce the risk of intracranial events?

### Rationale

The guideline committee was not able to make a recommendation on MR imaging in CYP with ADPKD or at risk of ADPKD. No relevant studies examining whether neurological imaging in CYP with ADPKD and a family history of intracranial events is associated with reduced cardiovascular morbidity compared with those who do not have neurological imaging, were identified. Three individual case reports of people under 18 years with ADPKD with intracranial events were found but did not meet inclusion criteria.

No consensus was achieved in 2 rounds of the Delphi survey; 61% of panellists agreed with the statement ‘Intracranial Magnetic Resonance (MR) imaging should be offered to CYP with ADPKD with a family history of intracranial aneurysm or sub-arachnoid haemorrhage’ (round 1), whilst 41% agreed with the statement ‘Intracranial Magnetic Resonance (MR) imaging should not routinely be offered to CYP with ADPKD even with a family history of intracranial aneurysm or sub-arachnoid haemorrhage’ (round 2). The lack of published data around screening in CYP < 18y was acknowledged and concern was noted with regard to thresholds for neurosurgical intervention if intracranial aneurysms were identified in CYP as a result of such testing.

A systematic review by Vlak et al. [29] estimated an overall prevalence of unruptured intracranial aneurysms (ICA) of 3.2% in a population without comorbidity. They calculated a prevalence ratio (PR) of 6.9 (95%CI 3.5–14) in patients with ADPKD compared to the population without comorbidity. Further analysis showed that the PR for patients with ADPKD and a family history of SAH or UIA was 2.0 (95%CI 0.5–7.4) compared with patients with ADPKD but no family history of SAH or UIA. The committee agreed that studies assessing the prevalence of SAH and ICA in CYP < 18y with ADPKD should be undertaken prior to making recommendations in this area.

## Lay summary

Autosomal Dominant Polycystic Kidney Disease (ADPKD) is thought to affect about 1 in 1000 people in the UK. ADPKD causes a progressive decline in kidney function, with kidney failure tending to occur in middle age. Children and young people with ADPKD may not have any symptoms. However they may have high blood pressure, which may accelerate progression to later stages of chronic kidney disease.

There is uncertainty and variation in how health professionals manage children and young people with confirmed or a family history of ADPKD, because of a lack of evidence. For example, health professionals may be unsure about when to test children’s blood pressure and how often to monitor it in the hospital clinic or at the GP. They may have different approaches in recommending scanning or genetic testing for ADPKD in childhood, with some recommending waiting until the young person is mature enough to make this decision his or herself.

This guideline is intended to help families affected by ADPKD by making sure that:

- health professionals with specialist knowledge in ADPKD offer you information on inheritance and potential benefits and harms of testing for ADPKD.
- the decision to test and the method of testing for ADPKD in children and young people is shared between you or your family and the health professionals
- blood pressure assessment is undertaken regularly in children and young people at risk of developing ADPKD
